# A Rapid Intelligent Screening of a Three-Band Index for Estimating Soil Copper Content

**DOI:** 10.3390/molecules30153215

**Published:** 2025-07-31

**Authors:** Shiyao Liu, Shichao Cui, Rengui Wang, Minming Han, Jingtao Kou

**Affiliations:** 1 College of Resources and Environment, Xinjiang Agricultural University, Urumqi 830052, China; l13993657830@163.com (S.L.); w15760153871@163.com (R.W.); m17899215745@163.com (M.H.); 18063553174@163.com (J.K.); 2 Xinjiang Planting Industry Green Production Engineering Technology Research Center, Urumqi 830052, China; 3 Xinjiang Engineering Technology Research Center of Soil Big Data, Urumqi 830052, China

**Keywords:** three-band spectral index, copper content, estimation model, CARS

## Abstract

Research has widely validated three-band spectral index as a simple, valid, and highly accurate method of estimating the copper content of soil. However, selecting the best band combination from hundreds of thousands, even millions of candidate combinations in hyperspectral data, is a very complicated problem. To address this issue, this study collected a total of 170 soil samples from the Aktas copper-gold mining area in Fuyun County, Xinjiang, China. Then, two algorithms including Competitive Weighted Resampling (CARS) and Stepwise Regression Analysis (STE) were applied to pick the bands from the original and first-order derivative spectra, respectively. A three-band index model was developed using the selected feature bands to estimate soil copper content. Results showed the first-order derivative spectrum transforms the spectral curve into a sharper one, with more peaks and valleys, which is beneficial for increasing the correlation between bands and copper content compared with the original spectrum. Moreover, integrating first-order derivative spectroscopy with CARS makes it possible to precisely identify key spectral bands and outperforms the dimensionality-reduction capabilities compared with the integration of STE. This strategy drastically reduces the time spent screening and is proven to have similar model accuracy, as compared to the individual group lifting method. Specifically, it reduces the duration of an 8 h task down to a mere 2 s. An intelligent screening of three-band indices is proposed in this study as a method of rapidly estimating copper content in soil.

## 1. Introduction

Copper mines have an important role in the contemporary economy and are essential for most industries and technological sectors. Copper is an excellent conductor of electricity and is widely used in power generation for electronics as well as electronics encased in communication equipment. It is also beneficial for construction and transportation, as well as manufacturing industries by facilitating infrastructure development and economic growth [1,2,3]. The demand for copper has increased greatly within the last few years, especially with the surging use of renewable energy and electric vehicles. The demand for these products has dramatically increased, and this has significantly enhanced attention on their extraction processes and resource-management methods.

Considering that surface and shallow mineral resources are depleting rapidly, there has been a major shift in focus from exploration of concealed minerals located beneath the Earth’s surface. Soil geochemical methods are frequently used to explore concealed mines [4,5,6,7,8,9,10,11,12,13,14]. The spatial distribution of metal concentrations of soil samples is analyzed by using these techniques to assist with the identification of geochemical anomaly zones. Geochemical anomalies allow refinement of the scope for exploration and delineation of the target mineralization areas. Nevertheless, this technique involves collecting a huge number of samples and measuring the amount of metal content in laboratory conditions, making it labor-intensive and time-consuming. However, noticeably, this approach is not applicable in some of the areas associated with adverse weather conditions. However, numerous studies have shown that hyperspectral remote sensing technology is a non-destructive, efficient, and comprehensive approach to estimate metal content in soil [15,16,17,18,19,20,21]. First, researchers extract spectral diagnostic indicators of the levels of metal content in soil spectra. Then, conventional statistical or machine learning methods are used to build quantitative estimation models. The constructed model of hyperspectral images related to aviation or aerospace can be utilized to obtain extensive and continuous spatial distribution information regarding soil metal content. This approach facilitates the identification of anomalous areas and the delineation of potential mineralization zones. Related studies illustrated that models constructed with spectral indices, especially with three-band combinations, have more effective diagnostic indicator features of that model as compared to those constructed with one dimensional spectral data [22,23,24,25,26]. The accuracy of these models is also higher than others, and they are more robust and generalized. Nevertheless, hyperspectral data involves a large number of spectral bands, ranging from hundreds to thousands. With this many bands given, there is an exceedingly large number of combinations of three bands. For example, with 100 bands, there are a total of 970,200 possible combinations. When the number of band data is 500, the number of potential band combinations explodes to 124,251,000. Therefore, it is a challenge to find a combination of three spectral bands necessary for accurate estimation of soil metal content.

At the same time, despite the large number of spectral bands present in hyperspectral data, these bands have a considerable degree of collinearity. As a result, not all bands contribute effectively to the estimation of metal content. Some bands may contain redundant information, indicating the presence of data redundancy. Therefore, the challenge is to narrow down the extensive range of spectral bands to identify the most useful. Subsequently, these bands can be utilized to construct a three-band index, thereby addressing the issue of excessive band combinations. If only 30 useful spectral bands can be identified, the number of potential three-band spectral combinations will decrease to 24,360. This figure represents one-thirtieth of the total possible combinations derived from 100 bands. Numerous studies have demonstrated that CARS and STE are effective strategies for selecting hyperspectral feature bands [27,28,29,30].

The objective of this study is to investigate the effectiveness of CARS and STE for screening feature bands and to develop a three-band spectral index based on the selected feature bands. Subsequently, we aim to establish a quantitative model that utilizes spectral indices that are strongly correlated to copper content. The goal is to evaluate the efficacy of this approach in determining whether it can not only generate high-precision estimation models but also reduce the number of bands, thereby simplifying three-band index screening. The developed model can possibly be further utilized toward hyperspectral imaging received through aerospace or airborne platforms. It would allow a quick outline of the soil copper anomaly in particular regions, the identification of possible mineralization destinations, and technical assistance in the exploration of hidden mineralization resources.

## 2. Materials and Methods

### 2.1. Research Area and Sampling Design

**Figure 1:**
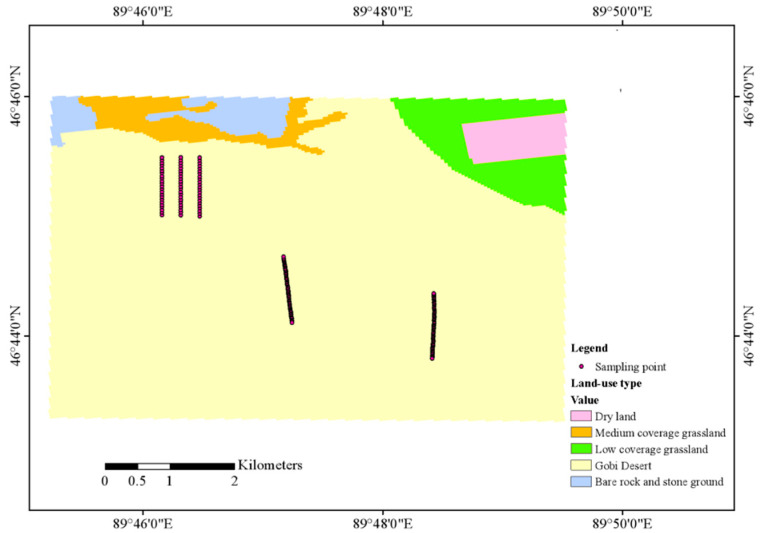
Spatial distribution map of sampling points.

The research area is in the Aktas copper-gold mining area, located in Fuyun County, Xinjiang Province, China. This area is in the Ertis mineralization belt, which is characterized by the presence of significant deep-seated faults. The Ertis deep major fault is in the north, while the Ulungu River deep major fault is in the south; additionally, the Kaiert-Ertai deep major fault is in the west. This area has a central position within the Shalbulak-Karatunk fold structure. The terrain of the study area is flat with large areas being covered with Quaternary unconsolidated deposits of Gobi Desert. The main components of these deposits are semi-cemented aeolian sands soil, alluvial–diluvial sand–gravel mixtures, or their combinations. The aeolian sandy soil consists of mainly silt type with the alluvial-diluvial gravels being highly varied in grain size—ranging between fine sand and boulders of more than 20 cm in diameter. This gravel is largely subangular in shape with poor sorting and low roundness. These loose overburden layers have an average thickness that lies between 3 and 5 m, but locally there are overburden layers that have reached more than 100 m. Most of the mining area is covered with Quaternary sediments, which poses a serious obstacle to mineral exploration. However, preliminary geophysical exploration combined with regional geological mapping and historical data suggests that the region still has high mineralization potential. Given the regional geological conditions, soil geochemical exploration methods with certain penetration capabilities have been identified as a highly promising exploration tool. To locate hidden ore bodies and explore the feasibility of using hyperspectral technology to quickly estimate soil copper content, a total of 170 soil samples were collected along five predetermined exploration profiles at depths ranging from 0 to 30 cm. Figure 1 shows the distribution of the sampling points. In each point identified, we take five subsamples, which are well distributed at the vertices of a pentagon in the circle of the radius of 5 m and merge into one composite sample.

In order to better present geochemical anomalies, the technique of partial extraction method was relied upon to reveal the amount of copper in the soil in this study. The flow of the particular process is as follows: after natural air-drying and oven-drying, soil samples collected in the wild are ground using an agate mortar and sieved through a 2 mm nylon sieve to obtain uniform particles. About 2.00 g of sieved representative soil sample are accurately weighed and placed in a 50 mL polypropylene centrifuge tube. Then 20 mL of prepared DTPA extraction agent is added, the centrifuge tube is sealed and placed on a constant temperature oscillator, and it is oscillated at 180 rpm for 2 h at 25° C. After the oscillation is completed, centrifugal separation is performed, the supernatant is filtered, and finally the ICP-MS (Agilent 8800 ICP-MS/MS, Santa Clara, CA, USA) method is used to determine the copper content in the soil. All samples were analyzed in triplicate, with an average relative standard deviation (RSD) of 5%.

### 2.2. Soil Spectral Measurement

**Figure 2:**
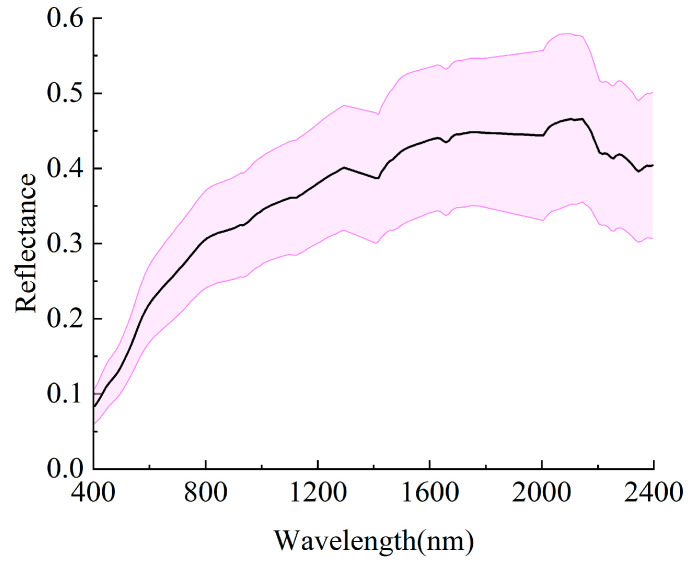
Mean and standard deviation of reflectance for the 170 soil samples after preprocessing. The black line represents the average reflectance of collected samples, and the pink area represents the standard deviation.

An ASD FieldSpec4 portable spectrometer (Malvern Panalytical Ltd., Malvern, UK, formerly Analytical Spectral Devices Inc., Westborough, MA, USA) is utilized to measure the reflectance spectrum of soil within the wavelength range of 400 to 2500 nm. To mitigate the impact of random noise, five spectral curves were obtained from each soil sample, and their average was computed to represent the final reflectance spectrum for each sample. To minimize the influence of instrumental and external noise, the wavelength bands ranging from 350 to 399 nm and from 2401 to 2500 nm were excluded. Furthermore, the bands within the ranges of 1300 to 1402 nm and 1793 to 2000 nm were also removed to reduce the effects of atmospheric water vapor. To mitigate data redundancy and address problems such as strong collinearity among adjacent spectral bands, this study applied an averaging technique on ten neighboring spectral bands to reduce dimensionality. The reflectance spectra of the soil samples collected after preprocessing are presented in Figure 2.

### 2.3. Feature Band Screening Method

CARS is important for hyperspectral regression. Typically, hyperspectral data is characterized by its high dimensionality and contains many redundancies, which poses major obstacles to accurate modeling and analysis. Adaptive resampling techniques are used to select the most typical features from high-dimensional datasets without losing too much information, thereby effectively reducing dimensionality. It improves the predictability of regression models, reduces the complexity of computation, and decreases the possibility of overfitting to some extent. Additionally, CARS performs well when solving multicollinearity, making it an ideal way to analyze complicated hyperspectral data. CARS also helps to minimize unnecessary features while enhancing model interpretability and providing a stronger basis for subsequent studies.

STE is one of the most frequently used statistical methods in hyperspectral regression that chooses independent variables, which improve the predicative performance of regressions. However, high data dimensionality and involved correlations in hyperspectral data make stepwise regression a good choice for selecting features very closely associated with the target. Secondly, it helps to reduce the effect of noise and redundant information. Stepwise regression helps researchers with finding the best predictive model by systematically adding or removing independent variables and improving the accuracy of final regression results. In addition, this method promotes the model interpretability and provides a more detailed understanding of the data of spectral and target variable relationships.

### 2.4. Development of a Three-Band Spectral Index

In this study, CARS and STE were initially implemented to conduct feature band selection from both the original spectra and their first derivative spectra. Following the identification of these feature bands, three-band indices were constructed based on the selected feature band. Subsequently, correlations between these indices and copper content in the soil were calculated, leading to the identification of spectral indices that demonstrated a high degree of correlation. The difference method is applied to compute the first derivative. The methodology for calculating the spectral index is presented in Table 1.

### 2.5. Estimation Model Construction and Evaluation

Order ranking of all the 170 samples of soil is performed on the basis of decreasing copper concentration. The ordered list is divided into contiguous triplets, where there are 56 triplets (168 samples) and 2 remaining samples. In all of the 56 triplets, two samples are taken at random and used in the training set, and the other single one is used in the testing set. Either one of the remaining sets is assigned randomly to the training set, and the other one is assigned to the testing set. The final training set has 113 samples, and the test set has 57 samples. A quantitative estimation model is developed utilizing spectral indices that exhibit a strong correlation with copper content, as identified from the training set. Subsequently, this model is applied to the testing set for performance evaluation. Determination coefficient (R^2^), root mean square error (RMSE), and the residual predictive deviation (RPD) were used for model performance evaluation. A larger R^2^ meant there was a smaller RMSE and that the model had a higher prediction accuracy. RPD values greater than 1.4 indicate that the model possesses certain predictive and generalization capabilities, whereas RPD values exceeding 1.8 demonstrate strong predictive and generalization abilities. Conversely, models with RPD values below 1.4 lack both predictive and generalization capabilities.

## 3. Results

### 3.1. Analysis of Copper Concentration in Soil Samples

**Figure 3:**
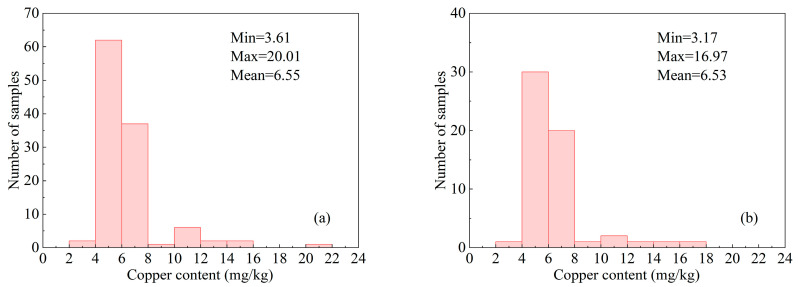
Statistical histogram of copper content in soil samples: (**a**) training set; (**b**) validation set.

The statistics concerning the copper content in soil samples obtained from both the training and validation sets are depicted in Figure 3. This figure provides a valuable visualization of the data-distribution characteristics inherent to both datasets, highlighting a significant degree of similarity between them. Upon analyzing the maximum, minimum, and average values for copper content across both sets, it is evident that they are close to one another, indicating consistency in the characteristics of the analyzed soil samples. Further examination through significance testing yielded a *p*-value exceeding the threshold of 0.05. This result suggests that there is no statistically significant difference between the two datasets, thereby reinforcing the reliability of our comparative analysis. Consequently, based on these findings, it is concluded that the utilization of data from the validation set to evaluate model performance developed from training set data is a sound and reliable approach.

### 3.2. Statistical Analysis of Selected Feature Bands

**Figure 4:**
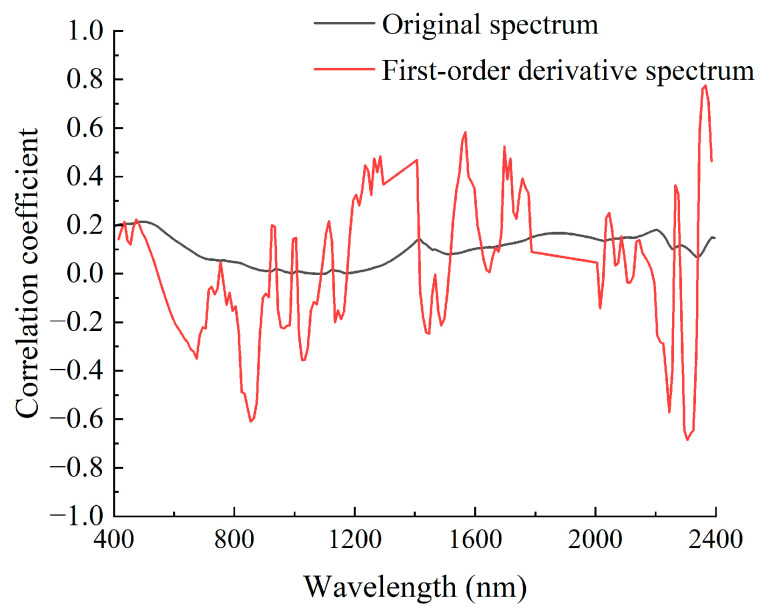
Correlation coefficients between different spectra and copper content.

The quantities and locations of the characteristic bands identified from both the original spectrum and its first derivative, utilizing CARS and STE, are presented in Table 2. Regardless of whether CARS or STE is applied, the number of feature bands selected from the first-order derivative spectrum is significantly smaller than that derived from the original spectrum, comprising only approximately half of the latter. Figure 4 shows that the correlation between the original spectrum and copper content is weak and exhibits slight variations in wavelength. In contrast, the correlation between the first-order derivative spectrum and lithium content demonstrates significant fluctuations in wavelength, characterized by numerous peaks and valleys. These spectral bands exhibit a substantial absolute correlation with copper content, with certain bands attaining values as high as 0.6. Compared to the original spectrum, first-order derivative spectra can enhance the correlation between specific bands and copper content, thereby serving as characteristic bands. As shown in Table 2, most of these bands have been selected through feature band screening. The results presented above indicate that, due to the ambiguity of the original spectral features, even when implementing feature band filtering methods, a large number of bands are selected. This leads to suboptimal dimensionality-reduction outcomes. In contrast, utilizing the first-order derivative spectrum can effectively reduce dimensionality by enhancing spectral features and integrating them with screening techniques.

### 3.3. Optimal Three-Band Spectral Index

Table 3 demonstrates that the maximum R^2^ value for the original spectrum is only 0.6209, while the maximum R^2^ value for the first derivative reaches 0.7651, with its minimum also attaining a value of 0.5910, which is comparable to the maximum of the original spectrum. This indicates that, in comparison to the original spectrum, the three-band index constructed from feature bands selected based on the first-order derivative spectrum is more strongly correlated with copper content. Table 3 illustrates that the minimum R^2^ value obtained using CARS was 0.5910, which surpasses the maximum R^2^ value of STE, recorded at 0.5717. This finding indicates that the spectral index derived from characteristic bands selected through CARS is more strongly correlated with copper content compared to that obtained via STE, regardless of whether it is based on the first derivative or the original spectrum. These findings suggest that integrating first-order derivative spectroscopy with CARS significantly enhances the capacity to extract spectral indices that correlate strongly with copper content.

### 3.4. Comparative Analysis of Models for Estimating Soil Copper Content

**Figure 5:**
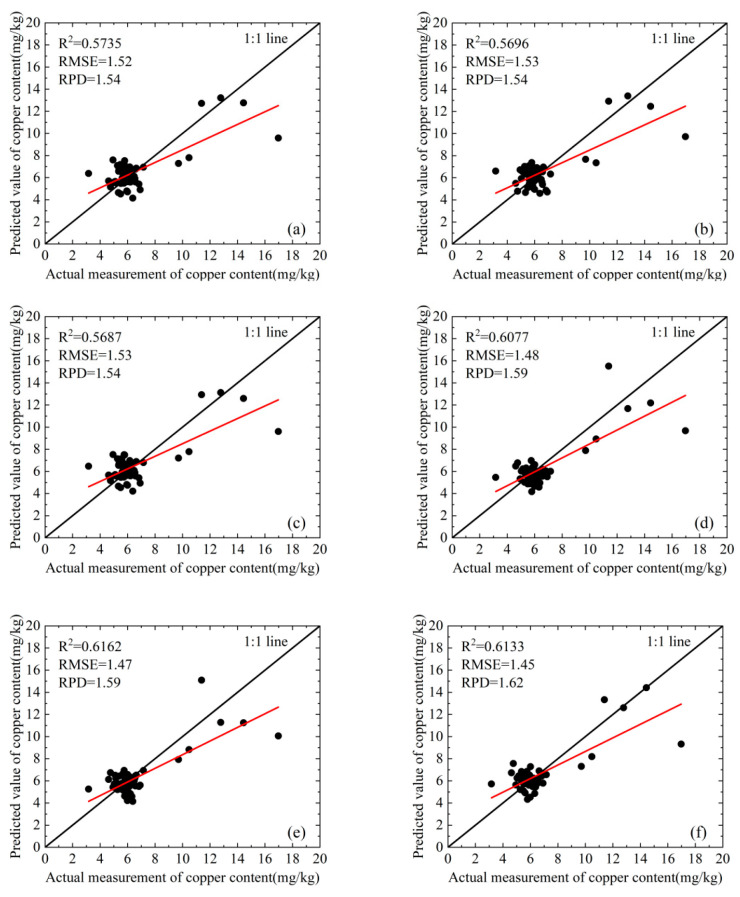
The performance comparison of the quantitative estimation model for copper content, which is constructed by selecting characteristic spectral bands through CARS to develop a three-band index: (**a**) original spectrum and TVI-1; (**b**) original spectrum and TVI-2; (**c**) original spectrum and TVI-3; (**d**) first-order derivative spectrum and TVI-1; (**e**) first-order derivative spectrum and TVI-2; (**f**) first-order derivative spectrum and TVI-3. The black scatter points represent paired data points of measured and estimated values, the black solid line is the fitting line between the two, and the red solid line is the 1:1 reference line.

**Figure 6:**
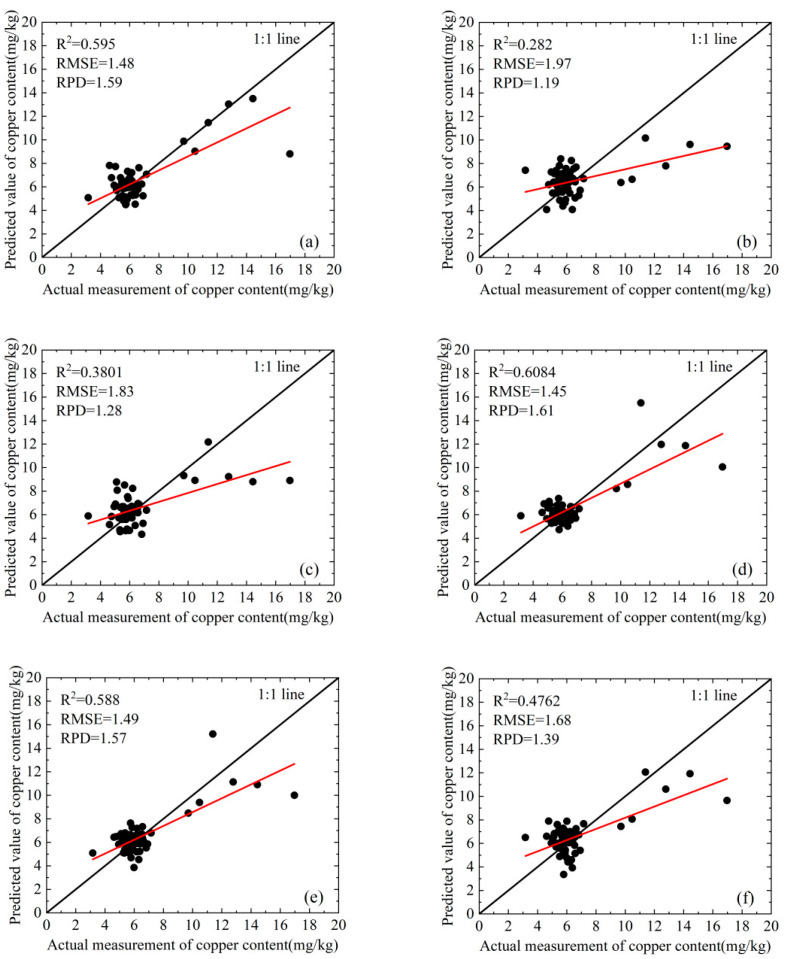
The performance comparison of the quantitative estimation model for copper content, which is constructed by selecting characteristic spectral bands through STE to develop a three-band index: (**a**) original spectrum and TVI-1; (**b**) original spectrum and TVI-2; (**c**) original spectrum and TVI-3; (**d**) first-order derivative spectrum and TVI-1; (**e**) first-order derivative spectrum and TVI-2; (**f**) first-order derivative spectrum and TVI-3. The black scatter points represent paired data points of measured and estimated values, the black solid line is the fitting line between the two, and the red solid line is the 1:1 reference line.

**Figure 7:**
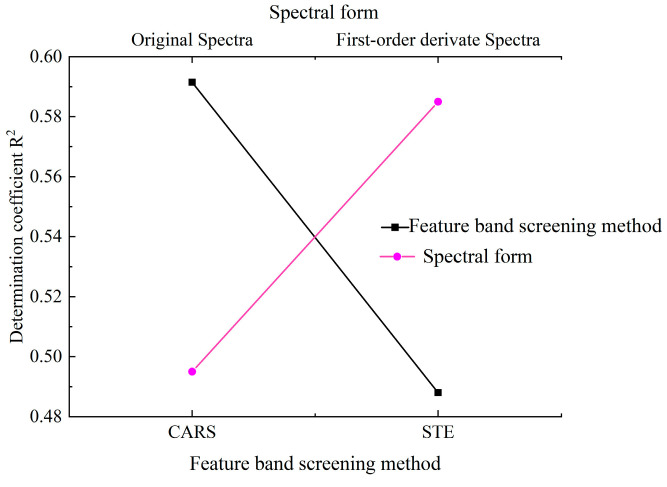
Comparison of the accuracy of copper content quantitative estimation models: between original and first-order derivative spectra, and between CARS and STE methods.

We develop a quantitative estimation model utilizing the three-band indices selected from the training set and apply this model to the validation set. The fitting results are presented in Figure 5 and Figure 6. These figures show that for the original spectrum, among the six models evaluated, the maximum value of the R^2^ for the validation set is only 0.595. In contrast, among the first derivative models, except for one model that achieved a value of 0.4762, all other models exhibit performance above 0.58, culminating in a maximum value of 0.6162. The average R^2^ values of six models for both the first derivative and the original spectrum are illustrated in Figure 7. This figure indicates that the average R^2^ value for the first derivative is approximately 0.6, which exceeds the 0.5 observed for the original spectrum. These results indicate that, compared to the original spectrum, models constructed using first-order derivative spectra demonstrate higher accuracy.

The average R^2^ value of the model developed using the three spectral indices selected by CARS and STE was statistically analyzed, as illustrated in Figure 7. The results indicate that the accuracy achieved through CARS for feature band selection and constructing a three-band index to develop a quantitative estimation model surpasses that obtained with STE. Consequently, the average R^2^ value increased from approximately 0.5 to 0.6. Compared to STE, the model constructed using CARS demonstrates greater stability. The R^2^ value for this model ranges from 0.5 to 0.6. In contrast, STE exhibits high volatility; models that perform well have an R^2^ close to 0.6, whereas those with poor performance exhibit an R^2^ of less than 0.3 (Figure 5 and Figure 6).

Considering the predictive capability and accuracy of the model, CARS combined with the first derivative and TVI-2, as well as CARS combined with the first derivative and TVI-3, represent two modeling approaches that demonstrate superior performance. The scatter plots illustrating the relationship between the measured and predicted values of the model are presented in Figure 5e,f. This analysis demonstrates that the predicted values are symmetrically distributed around the 1:1 line, indicating that there is no significant overall overestimation or underestimation. The RPD of the model is greater than 1.4 but less than 1.8, suggesting that it possesses predictive capability. However, this ability is not particularly strong.

## 4. Discussion

The accuracy, rapidity, and non-destructiveness of hyperspectral technology have been thoroughly investigated, and the advantages of this approach in estimating metal content in soil have been fully demonstrated. While having lots of spectral bands contained in hyperspectral data has many merits for detecting diagnostic indicators of soil metal content, it has the disadvantages of redundant data and high collinearity. If these issues are not properly addressed, the resulting estimation model will likely be overfitted. In this case, although the model performs well on the training set, the accuracy will drop very significantly if the validation set is sufficiently independent from the training data. To address this issue, several researchers currently implement screening methods to identify characteristic bands from both the original and transformed soil spectra [31,32,33,34]. Using the identified characteristic bands, they then develop the quantitative estimation models. This strategy was effective, as they demonstrated that it both reduces model complexity and increases both its robustness and generalization capability. The spectral index utilizes multiple types of band information to calculate indices, improves efficiency in information extraction, and improves the ability of denoising, while strongly resisting the interference of environmental factors. The spectral index addresses the complex nonlinear relationship between soil metal content and spectral characteristics and improves prediction accuracy. Another advantage is that designing specific indices results in more adaptable and flexible research. Therefore, the model is simplified, and the results are made interpretable. The spectral index provides significant advantages over the modeling of soil metal content based on individual spectral bands, which has contributed to its extensive number of contemporary applications. However, selecting the optimal combination of bands from a multitude of possible combinations is extremely difficult. Currently, most studies adopt a straightforward and somewhat brute-force approach by individually calculating the correlation between each possible combination of spectral bands and metal content. Ultimately, the combination with the highest correlation is selected as the optimal band combination. Due to the small number of band combinations, this approach may be suitable for two-band spectral indices. However, the number of possible three-band combinations is vast, often reaching into the millions. This makes it exceedingly complicated to identify the optimal combination. Continuing with a step-by-step approach would be both time-consuming and labor-intensive. For instance, in this study, the first derivative samples of soil exhibited 168 spectral bands, resulting in a total of 4,657,296 possible combinations of three bands. The process of selecting the optimal combination required approximately 30,119.77 s, which is nearly equivalent to 8 h. Therefore, there is an urgent need for innovative strategies to efficiently screen for the optimal band combination. Many studies have revealed that CARS and STE are feasible methodologies for screening feature bands [35,36,37,38,39]. Therefore, this study initially applies these methods to identify the characteristic bands, selecting the spectral bands that are relevant for assessing copper content. Subsequently, three-band spectral indices are constructed based on the selected bands. The results indicate that compared to STE, CARS is more effective. The R^2^ between the optimal three-band index that was selected through the group method and the copper content is about 0.77; the result through CARS is 0.76, and there is a small difference. CARS decreases processing time from around 8 h to about 2 s, which is significant. These results show that CARS is an effective method to first screen characteristic bands and then to construct a three-band vegetation index based on these bands. Nevertheless, when implementing this method, it is essential to carefully select suitable spectra for the screening of feature bands. The results of this study show that derivative spectra are capable of significantly sharpening the spectral curve, producing multiple distinct peaks and valleys, magnifying the weak signal features, and enhancing correlations between the spectral bands and copper content in comparison with the original spectrum. This finding is consistent with previous research [40,41,42,43,44]. Additionally, this study shows that the first-order derivative spectra incorporated with CARS allows for more efficient dimensionality reduction compared to the original spectra. Consequently, this study found that the integration of first-order derivative spectroscopy, CARS, and a three-band spectral index would contribute to high-precision, rapid, and non-destructive estimation of soil copper content.

This paper suggests a rapid detection model, hyperspectral-based, in the determination of anomalies of copper content in soil. The next research stage will include the use of the model in mining regions to outline possible mineralization targets depending on the revealed copper anomalies. They will then be followed up by field investigations like drilling programs to confirm the presence of ore bodies within these targets and to ascertain important factors like ore-body thicknesses, grade, and others. This validation will finally examine how the hyperspectral model of detection can be practically useful in exploration of minerals.

## 5. Conclusions

This study aims to apply two methods, STE and CARS, to identify characteristic spectral bands from soil spectra. Based on the selected bands, a three-band index is constructed, followed by the establishment of a model for estimating copper content. The findings indicated that, in comparison to STE, CARS demonstrates superior effectiveness. It precisely extracts essential characteristic bands while simultaneously reducing dimensionality, which significantly lowers the number of bands from 168 to 12. The correlation between the three-band index derived from the 12 selected bands and copper content exhibited only a minor reduction when compared to the three-band index determined via cluster analysis. However, the screening time was significantly reduced from 8 h to 2 s. This study also found that, compared to the original spectrum, the three-band index constructed using characteristic bands selected from the first derivative spectrum through CARS exhibits a stronger correlation with copper content. Furthermore, the accuracy of the quantitative model is enhanced, and its predictive capability is significantly improved. This study demonstrates that the integration of CARS, first derivative analysis, and three-band indices can yield highly accurate estimations of soil copper content. Furthermore, this research proposes an innovative approach for the intelligent screening of three-band indices to rapidly estimate copper content in soil. Lastly, the study proposes an approach that will allow the fast and proper detection of copper anomalies in the soil using hyperspectral technology that will give technical assistance in exploring hidden mineral deposits.

## Figures and Tables

**Table 1:** Calculation formula of three-band spectral index.

Name of Spectral Index	Calculation Formula
TVI-1	(S_1_ − S_2_)/S_3_
TVI-2	(S_2_ + S_3_)/S_1_
TVI-3	(S_1_ − S_2_)/(S_3_ − S_2_)

S1, S2, and S3 represent spectral values at bands 1, 2, and 3, respectively.

**Table 2:** Statistical analysis of feature bands selected using different methods.

Spectral Type	Screening Method	Number of Bands	Position of Bands (nm)
Original spectrum	CARS	26	404.5, 674.5, 734.5, 774.5, 784.5, 824.5, 1164.5, 1194.5, 1204.5, 1214.5, 1224.5, 1264.5, 1274.5, 1417.5, 2155.5, 2165.5, 2175.5, 2185.5, 2215.5, 2225.5, 2305.5, 2315.5, 2325.5, 2335.5, 2345.5, 2395.5
Original spectrum	STE	13	434.5, 444.5, 454.5, 464.5, 564.5, 604.5, 1014.5, 1034.5, 1094.5, 1194.5, 1214.5, 1294.5, 1417.5
First-order derivative spectrum	CARS	12	434.5, 604.5, 804.5, 884.5, 914.5, 1024.5, 1447.5, 1647.5, 2155.5, 2235.5, 2275.5, 2365.5
First-order derivative spectrum	STE	5	634.5, 724.5, 1024.5, 2305.5, 2365.5

**Table 3:** Three-band index with high correlation to copper content.

Spectral Type	Screening Method	Spectral Index Type	Determination Coefficient R^2^
Original spectrum	CARS	TVI-1	0.6093
Original spectrum	CARS	TVI-2	0.6087
Original spectrum	CARS	TVI-3	0.6209
Original spectrum	STE	TVI-1	0.3752
Original spectrum	STE	TVI-2	0.3915
Original spectrum	STE	TVI-3	0.5717
First-order derivative spectrum	CARS	TVI-1	0.7514
First-order derivative spectrum	CARS	TVI-2	0.7651
First-order derivative spectrum	CARS	TVI-3	0.5910
First-order derivative spectrum	STE	TVI-1	0.7592
First-order derivative spectrum	STE	TVI-2	0.7654
First-order derivative spectrum	STE	TVI-3	0.5257

## Data Availability

The original contributions presented in the study are included in the article; further inquiries can be directed to the corresponding author.
